# Supplementary material for: Adaptive and context-aware volumetric printing
Source: Nature. 2025 Sep 3;645(8079):108–14. doi: 10.1038/s41586-025-09436-7 (PMC12408377; doi:10.1038/s41586-025-09436-7)
Supplement: Supplementary file 1 — The Supplementary Information file contains: Materials and Methods 1–23, Supplementary Figs. 1–20, legends for Supplementary Videos 1–5 and Supplementary References. [file 41586_2025_9436_MOESM1_ESM.pdf]

nature portfolio

https://doi.org/10.1038/s41586-025-09436-7

Supplementary information

Adaptive and context-aware volumetric printing

In the format provided by the authors and unedited

Supplementary Materials for

Adaptive and Context-Aware Volumetric Printing

Sammy Florczak[1,2], Gabriel Größbacher[1], Davide Ribezzi[1], Alessia Longoni[1], Marième Gueye[1], Estée Grandidier[1], Jos Malda[1,2], Riccardo Levato[1,2]*

[1] Department of Orthopaedics, University Medical Center Utrecht, Utrecht University; Utrecht, 3584 CX, The Netherlands.

[2] Department of Clinical Sciences, Faculty of Veterinary Medicine, Utrecht University; Utrecht, 3584 CT, the Netherlands.

* Corresponding author. Email: r.levato@uu.nl

The PDF file includes:

Materials and Methods S1 to S23
Supplementary Figures 1 to 20
Supplementary Videos 1 to 5
References

Supplementary Methods

<u>S1. Cost-effective design light sheet illumination</u>

A homogenized light sheet source was constructed using readily available off-the-shelf components. The accompanying price list corresponds to a simplified configuration used in our system, operating at 532 nm with a single laser source. Since the Powell lens introduces controlled divergence along the long axis of the light sheet to achieve uniform intensity, an additional cylindrical lens can be used downstream to partially recollimated the beam (not used in our system). Doing so may reduce angular spread and helps maintain a thinner sheet over a longer propagation distance. Alternatively, a cylindrical lens can be used in place of the Powell lens for simpler, lower-cost implementations, at the expense of some uniformity. When paired with a suitable camera, imaging lens, and appropriate emission filters, this configuration enables an effective fluorescence imaging system at a significantly reduced cost compared to conventional setups.

Item	Part Number	Supplier	Qty.	Unit Price (€)	Total (€)
Laser Source					
532nm 50mW Laser	RLDD532-50-3	Roithner GmbH	1	313.00	313.00
Optics					
f=50mm Cylindrical Lens	LJ1695L2-A	Thorlabs	1	151.17	151.17
f=125mm Plano-convex lens	LA1986-A-ML	Thorlabs	1	47.52	47.52
f=-50mm Plano-concave lens	LC1715-A-ML	Thorlabs	1	45.90	45.90
30° Powell Lens	43-473	Edmund Optics	1	242.00	242.00
Optomechanics					
Small V-clamp	VC3C/M	Thorlabs	1	41.04	41.04
Kinematic Mirror Mount	KM100	Thorlabs	2	38.04	76.08
Lens Mount	LMR1/M	Thorlabs	2	14.97	29.94

Cylindrical Lens Mount	CH2B	Thorlabs	1	70.47	70.47
Large V-Clamp	VC3C/M	Thorlabs	1	41.04	41.04
				**Total**	1058.16

As complementary information to the above-mentioned components that can be used to build the same light sheet imaging system used in this study, we would also like to highlight that additional blueprints for custom-made, affordable light sheet imaging systems are also accessible through more peer reviewed publications[1].

S2. Light sheet imaging pre-processing

To mitigate alignment errors, two preprocessing steps were implemented during image acquisition. First, a horizontal crop was applied to ensure precise centring of the vial within the image frame, thereby minimizing pixel-scale displacement errors that could arise from minor horizontal camera misalignment. Additionally, a vertical crop was employed to delineate the bounds of the maximum projection height (0 mm -14 mm in our system), corresponding to the 1:1 imaging (projection) of the DMD onto the vial centre. This was also important for defining the polar coordinates based on the pixel row and column index.

S3. Additional hardware details for experimental implementation

Orthogonality between the light sheet, imaging, and post-DMD projection optical paths was achieved by integrating all components into a cage system centred upon a precision cage cube. To ensure absolute congruence between the printed part and imaged features, as well as setting a datum for successive prints, the rotational stage holding the sample required homing functionality. In our system we utilized a rotational stage (Newmark RB-90-3-U, USA) with an incorporated homing sensor, although this alignment could alternatively be achieved with an absolute encoder or by using the differential encoder's index channel.

S4. Pipeline for feature coordinate registration and data processing in GRACE

A MATLAB (MathWorks, USA) script was developed to handle key functions, culminating in the export of the coordinate table. In our implementation of GRACE, the following steps were performed:

1. Initialization**:**
    a. Set Imaging and Processing Parameters:
        i. Angular step size: $\Delta\theta$
        ii. Scan Range: Θ_{total} (typically 0 to π, or 0 to 2π)
        iii. DBSCAN parameters (if clustering is required):
            - Minimum number of neighbouring points to form a cluster: *minPts*
            - Neighbourhood radius: ε
        iv. Image cropping parameters to ensure vial centrality.
        v. Feature type (i.e. multiple populations).
    b. Hardware Initialization
        i. Home the rotational stage to establish a reference datum.
        ii. Initialize the camera for image acquisition.
        iii. Initialize the light sheet.
2. Image Acquisition:
    a. For each angular position θ_i in the range $[0, \Theta_{total}]$ with step size $\Delta\theta$:
        i. Rotate the stage to angle θ_i.
        ii. Capture the image frame $I(\theta_i)$
        iii. Store $I(\theta_i)$ sequentially.
3. Feature Isolation. To extract useful information from the polar stack, thresholding and segmentation is performed to isolate features-of-interest from the background.
    a. For each captured image $I(\theta_i)$:
        i. Apply thresholding to isolate features:

$$I_{binary}(\theta_i) = 1, \quad if \quad I(\theta_i) \geq T$$

or

$$I_{binary}(\theta_i) = 0, \; if \quad I(\theta_i) < T$$

        ii. Store binary images $I_{binary}(\theta_i)$.

4. Coordinate Transformation:
    a. For each pixel with value 1 in $I_{binary}(\theta_i)$ at position (r_{ij}, z_{ij}):
        i. Convert polar coordinates (r_{ij}, θ_i, z_{ij}) to Cartesian coordinates (x_{ij}, y_{iz}, z_{ij}):

$$x_{ij} = r_{ij} \cos \theta_i$$

$$y_{ij} = r_{ij} \sin \theta_i$$

    b. Aggregate all (x_{ij}, y_{iz}, z_{ij}) to point cloud *P*.
5. Radial Sampling Homogenization:
    a. To homogenize radial sampling frequency, for each (x_{ij}, y_{iz}, z_{ij}) in *P*:
        i. Calculate radial distance $r_{ij} = \sqrt{x_{ij}^2 + y_{ij}^2}$
        ii. Determine culling probability $p(r_{ij})$ proportional to r_{ij}.
        iii. Randomly keep or cull points based on $p(r_{ij})$.
6. Clustering and Centroid Determination (if required):
    a. If clustering is desired (i.e. for identifying and labelling discrete features):
        i. Apply the DBSCAN algorithm to *P* with parameters ε (neighbourhood search radius around the point), and *minPts*, resulting in clusters $C_n = \{c_1, c_2, \ldots, c_{N_n}\}$ for points $n = 1,2, \ldots, N$ belonging within that cluster.
    b. For each cluster C_n, compute the centroid $(\bar{x}_n, \bar{y}_n, \bar{z}_n)$:

$$\bar{x}_n = \frac{1}{N_n} \sum_{(x_{ij}, y_{iz}, z_{ij}) \in C_n} x_{ij} \quad (1)$$

$$\bar{y}_n = \frac{1}{N_n} \sum_{(x_{ij}, y_{iz}, z_{ij}) \in C_n} y_{ij} \quad (2)$$

$$\bar{z}_n = \frac{1}{N_n} \sum_{(x_{ij}, y_{iz}, z_{ij}) \in C_n} z_{ij} \quad (3)$$

where N_n is the number of points within the nth cluster.

c. Determine the radius r_k (if necessary) as the 95th percentile of distances from the centroid to the points in C_k :

$$r_k = \boldsymbol{P}_{\mathbf{95}}\sqrt{(x_{ij} - \bar{x}_n)^2 + (y_{iz} - \bar{y}_n)^2 + (z_{ij} - \bar{z}_n)^2}$$

7. Data Export:
   a. If using raw data (e.g., for encapsulation, wrapping channel networks around arbitrarily shaped features, or for auto-alignment):
      i. Export point cloud P to a table (.csv) file containing centroid coordinates $(\bar{x}_n, \bar{y}_n, \bar{z}_n)$.
   b. If using clustered data:
      i. Export file containing centroid coordinates $(\bar{x}_n, \bar{y}_n, \bar{z}_n)$, Cluster radii r_n, and cluster labels n.
8. Repeat for Multiple Spectral Channels (If applicable):
   a. Repeat Steps 2–7 for each spectral channel to obtain separate datasets P_s for each channel s.

S5: Light sheet comparisons and benchmarking

In our work we primarily explored the use of externally generated light sheets to perform feature mapping. An alternative, strategy is also to simply leverage the volumetric printing illumination source itself by encoding a single central column of on-state pixels onto the DMD, and projecting that onto the axis of the printing volume (Supplementary Fig. 1a,b) . Though inefficient in terms of power-delivery - as much of the light is lost to the off-state pixels - and with the potential to cause unintended crosslinking of the sample (unless a different wavelength or short exposures times are used), it non-the-less provides a potentially useful alternative source for reconstructing volumes that could be used with GRACE. We validated this by demonstrating the use of the DMD-based light sheet to reconstruct a volume laden with fluorescent beads and benchmarking it by comparing the diameters with the same reconstructed volume generated using the 450nm, 532nm, and 650nm external light sheet sources.

To perform this, green, fluorescent polyethylene beads of diameters 125-150μm and 425-500μm (UVPMS-BG-1.0 125-150, and UVPMS-BG-1.0 425-500 respectively; Cospheric, USA) were first suspended within a 5% solution of gelatine and distilled water. The two samples, containing either the larger or smaller particles, were imaged with each light sheet source (405nm DMD, and the 450nm, 532nm and 650nm external light sheets), to generate a 3D reconstruction of each sample (Supplementary Fig. 1c,d). Scanning was performed with a 550-600nm bandpass filter for all but the 650nm external light sheet, as at this longer wavelength no fluorescent excitation of the beads was possible. Instead, the back reflected signal of the 650nm source was used. The 3D reconstructions were then generated, and particle diameters were randomly sampled. The results were also compared with diameters obtained via brightfield microscopy (Leica DMi8) (Supplementary Fig. 1e,f).

S6: Preparation of alginate microparticles

Alginate microparticles were produced by dissolving sodium alginate (alginic acid sodium salt, MilliporeSigma) in deionized water at 2% w/v concentration. A crosslinking solution was prepared by dissolving $CaCl_2$ (Sigma-Aldrich) in DI water, reaching a final concentration of 100 mM. An in lab-made platform was purposely built for microparticles production, consisting of a coaxial needle system (RegenHu, Switzerland), where the inner needle was used for airflow and the outer for alginate flow. Two Inkredible bioprinters (Cellink, BICO Company, Sweden) were used to regulate the pressure for both the airflow and alginate flow rate. A collection bath, filled with 100 mM calcium chloride solution, was utilized for the collection and crosslinking of microparticles. The collection bath was placed below the coaxial needle and on top of a magnetic stirrer, which was used to homogeneously stir the solution in the collection bath. Once the setup was built, alginate hydrogel solutions with different fluorescent dyes (Cy-5 and Cy-3.5) were prepared and loaded into 3 mL cartridges, which were finally connected to the coaxial needle. The air pressure and flowrate were regulated to produce different sizes of microparticles. After generating the microparticles, they were strained and transferred into a 50 mL Falcon tube and washed using deionized water. A fluorescent microscope (Leica Microsystems SP8, Germany) was used for imaging and characterization of microparticle size.

S7: Printability of convoluted channels

An assessment of attainable print resolution in the XY axis was undertaken by volumetric printing of small cylindrical structures of 4 mm external diameter, 7mm length, and with internal target apertures ranging between 0.5 mm to 0.1mm. The tubular structures were printed for a duration until visibly crosslinked under Schlieren imaging, then washed in warm PBS. This was performed in triplicate. The internal diameters of the printed constructs were then imaged transversely using the linear light sheet and analysed in ImageJ (Supplementary Fig. 9). It can be seen that on our system, deviations in dimensional accuracy began to occur with channels at 0.2mm and smaller, with the internal channel diameter measuring consistently less than the target size below that threshold. For this reason, we focused primarily on channel sizes greater than 0.2mm for our experiments throughout this work.

To assess the printability of complex constructs, convoluted spherically wrapping channel networks were printed at a fixed target channel diameter of 0.4mm. Models were generated parametrically in Grasshopper by defining a series of randomly positioned "target nodes" to which channels would be targeted towards. This was generated within a spherical region of interest in a cylindrical bulk scaffold with both an inlet and outlet (Supplementary Fig. 10a, b).

S8: Parametric model preparation

Grasshopper enables the creation of parametric models within the Rhino3D ecosystem, offering a robust array of mathematical operations and, crucially, allowing for seamless synchronization with external data sources. This integration facilitates dynamic model adjustments based on input from the imaging system. By leveraging Grasshopper, we prepared a diverse array of parametric models to be driven adaptively by our processed data. Importantly, unlike traditional modelling paradigms that require each model to be built from scratch, the parametric nature of these models allows for instant modification of any structural properties. This approach enables rapid, on-the-fly customization based on specific requirements or experimental conditions, significantly streamlining the design process.

For the encapsulation of single points (as demonstrated in Fig. 2c) where features were assumed to be spherical (such as with the alginate microspheres) the centroid and radii data were first

imported and synchronized within GH. Spherical geometries with radii corresponding to the feature radius plus an additional desired offset (i.e. shell thickness) were then positioned around each centroid, thus enabling the simple encapsulation of these features with spherical shells upon printing (Supplementary Fig. 4**)**.

Interconnection of spherical features (as demonstrated in Fig. 2b) was achieved as in the encapsulation regime, but with the addition of generating linear paths between the centroid coordinates using the "Proximity3D" and "Curve to Volume" functions respectively. These paths were then converted to 3D geometry using the 'multi-pipe' function within GH (Supplementary Fig. 3).

Organic vessel-like geometries, including solitary grazing channels, spherically wrapped channel networks (as demonstrated in Fig. 2a,d,e), as well as channel formation around arbitrarily shaped features (including the EmVP printed toruses) were all generated in GH using the same general concept (see Supplementary Fig. 2 for a simplified GH definition flowchart). This broadly involved the following:

1. Data Import and Synchronization: Coordinate or point-cloud data P was imported and synchronized within the GH definition.
2. Bulk Structure: A cylindrical construct was defined as the bulk structure for the model.
3. Region of Interest Delineation: A region of interest was defined to prevent channels from being generated too close to the walls of the bulk structure.
4. Distance Offset from Feature: An offset region was defined around the coordinate data, representing the surface targeted for channel formation.
5. Random Point Distribution: The remaining volume was populated with randomly distributed 3D points.
6. Network Generation: The 'Proximity3D' function was employed to connect the most proximal points, creating a random sub-network of potential pathways for channel formation. This network spanned both the bulk volume, and the surface offset regions around features of *Step 4*. This also included the placement of inlet and outlet paths.
7. Channel Endpoint Definition: Target starting and end points for all channels were defined.

8. Conditional Relationship Implementation (if required): Additional constraints and relationships were incorporated based on spectral data, volumetric position, feature labels/indices, or morphological characteristics.
9. Path Optimization: The shortest walk algorithm was applied to determine optimal paths along the line network.
10. Geometry Conversion: Resultant paths were converted into branched geometries using either the "DENDRO" plugin or built-in "Polypipe" functions, forming vessel-like structures around the features. This resulted in a positive channel structure that could be subtracted from the bulk cylindrical structure.
11. Negative Space Creation: Boolean subtraction was performed on the bulk cylindrical structure to generate negative channel spaces.

The parametric nature of these models allows for automatic recalculation of geometry when new data is introduced. Upon implementation, the geometry automatically recalculates, generating bespoke models without further user intervention. Key parameters—such as encapsulation thickness, vessel diameter, and various relational attributes—can be adjusted on-the-fly, allowing for rapid design iterations.

S9: Perfusability of GRACE constructs

3D models (in STL format) were generated with GRACE. The channel diameters were set to 500, 400 and 300 μm respectively and the exported CAD files for printing were printed using two hydrogels. The first hydrogel consists of 5% (w/v) Poly(ethylene glycol) diacrylate (PEGDA, Mn 700, Sigma-Aldrich), and 0.1% (w/v) lithium phenyl(2,4,6-trimethylbenzoyl)phosphinate (LAP, Tokyo Chemical Industry). Due to the low viscosity of PEGDA, to prevent sedimentation during the tomographic volumetric printing step, the resin was supplemented with 3% (w/v) gelatin (from porcine skin, 175 Bloom, Sigma-Aldrich), allowing for thermal gelation. The second hydrogel consisted of 10% (w/v) GelMA (as described previously) and 0.1% (w/v) LAP. Samples were printed in a tomographic volumetric printer as previously described (PEGDA: light dose of 190 mJ for the 500 μm, 185 mJ for the 400 and 300 μm structure respectively; (GelMA: light dose of 150 mJ for all channel sizes). Samples were mixed with 15 μL mL-1 Cy3.5–PEG–SH (Mw = 5 kDa) (Biopharma PEG) to increase contrast when imaging with light sheet microscope. Lightsheet

imaging was performed using a 532 nm laser, and the light sheet image stacks were imported into Fiji (ImageJ v1.54p), and the resulting image stacks were pseudo flat field corrected using the ImageJ plugin BioVoxxel, and subsequently imported into Matlab (MathWorks 2024a), with which a 3D image was rendered using the Volume Viewer app. Flow visualizing was performed using a bright field stereo microscope (Olympus, SZ61) to record the perfusion of the printed channels with Alcian Blue. The diameter of the vessels was measured from light sheet cross-sections crossing the middle of a channel, analyzing at least twelve randomly selected vessels per sample.

S10: Optimizing pillar phantom for shadow correction

We conducted a systematic stochastic optimization to determine an optimal configuration of occlusions that would maximize the demonstrable efficacy of GRACE workflow for shadow correction using the OSMO algorithm. This process involved a Monte Carlo-based parametric sweep, varying both the number (*n*), diameter (*d*), and spatial distribution of 2D occlusions within a simulated volume area.

For each iteration i, we computationally generated a random distribution of pillars $Q_i = \{q_1, q_2, \ldots, q_n\}$, where q_j represents the 3D coordinates of the jth pillar. We then applied the OSMO algorithm to optimize the tomographic back projections. The improvement was quantified using two metrics. First, the Jaccard index (*J*), given as:

$$J(A, B) = \frac{|A \cap B|}{|A \cup B|}$$

where A and B are the reconstructed and target volumes, respectively; was used to measure the similarity between the target, and the resultant computational reconstruction as determined by the best attainable binary dosage threshold. Secondly, the Bhattacharyya coefficient (BC):

$$BC(h_1, h_2) = \sum_x \sqrt{h_1(x) \cdot h_2(x)}$$

where h_1 and h_2 are normalized intensity histograms. The Bhattacharyya coefficient compared the histograms between the in-target and out-of-target dose reconstructions – with a lower coefficient indicating less overlap and thus better contrast. These were performed in the context of the cog-like print target (Fig. 3b,c). The process was repeated across a range of pillar quantities to identify the configuration for which J and BC were minimized. The identified optimal configuration was subsequently physically implemented to experimentally validate the workflow for shadow mitigation under real-world conditions.

Additionally, a global parametric sweep was performed, focusing on a range of diameters from 0.1mm to 1mm, and occluder numbers between 1-30, with the cog geometry serving as the reconstruction target, and maintaining a fixed vial diameter of 15mm to mimic the real-world print volume geometry. This analysis allowed systematic evaluation across a range of parameters, providing insights into how these variables impact overall performance.

S11: Shadow correction data processing pipeline for vertical pillar occlusions

Cluster detection was employed to identify distinct point clouds clusters C_n for each pillar, where $C_n = \{c_1, c_2, \ldots, c_{N_n}\}$ for points $n = 1,2,\ldots,N$ belonging within that cluster, and $c_k = (x_{ij_n}, y_{iz_n}, z_{ij_n})$ represents individual points in vial-space coordinates. The centroid $\overline{c_n} = (\bar{x}_n, \bar{y}_n, \bar{z}_n)$ of each pillar's point cloud was calculated as described previously using Eq. 1-3. To account for potential angular tilt of the phantom, which may arise from minor surface irregularities at the base of the vial, we employed principal component analysis (PCA) on the point cloud data. Specifically, we calculated the eigenvectors of the covariance matrix to determine the primary axis of each pillar. This approach allowed us to accurately measure and compensate for any deviation from perfect verticality, ensuring precise reconstruction of the pillar geometries within Grasshopper. The covariance matrix Σ_i for each pillar was computed as:

$$\Sigma_i = \frac{1}{N_n}\sum_{k=1}^{N_n}(p_k - \overline{c_n})(p_k - \overline{c_n})^{\mathrm{T}} \quad (4)$$

where n_i is the number of points in the ith pillar's cluster. The eigenvectors $\boldsymbol{v}_1, \boldsymbol{v}_2, \boldsymbol{v}_3$ of Σ_{i} were calculated, with $\boldsymbol{v}_1$corresponding to the largest eigenvalue, and thus representing the primary axis of the pillar. The pitch (θ) and yaw (ϕ) angles were derived from $\boldsymbol{v}_1 = (v_x, v_y, v_z)$ as:

$$\theta = \operatorname{acos}\frac{v_z}{\|\boldsymbol{v}_1\|} \tag{5}$$

where $\|\boldsymbol{v}_1\| = \sqrt{{v_x}^2 + {v_y}^2 + {v_z}^2}$, and

$$\phi = \operatorname{atan2}(v_y, v_x) \tag{6}$$

These values (centroids, pitch, and yaw) were then synchronized with a pre-prepared GH definition, allowing for parametric generation of corresponding cylindrical surfaces at the correct real-world positions of the occluding pillars (Supplementary Fig. 5c).

S12: Analysis of shadow corrected prints

To quantifiably compare the corrected and uncorrected spherical prints, the following metrics were used: *i)* root-mean-square error (RMSE); and *ii)* sphericity (ψ). To perform this, the resultant light sheet scans of the samples were segmented, and used to reconstruct polygon meshes of the prints. The RMSE is based on the deviation from the ideal target geometry, expressed as:

$$RMSE = \frac{1}{N}\sum_{i=1}^{N} d_i^2 \tag{5}$$

where N is the number of points sampled and d_i is the deviation of that point from the corresponding point on the target geometry. Sphericity is a measure of how closely the object resembles a sphere, and is defined as the ratio of the surface area of a sphere (with the same volume as the object) to the surface area of the object:

$$\psi = \frac{\pi^{\frac{1}{3}}(6V_p)^{\frac{2}{3}}}{A_p} \tag{6}$$

where V_p is the volume, and A_p the surface area of the object.

S13: Shadow correction for vascular structures printed within an occluding stent-like cage.

To investigate the printability of negative features and hollow channels in the context of shadow correction, the auto-alignment based workflow as described for the ball-in-cage model, was used to volumetrically print around an occlusive SLA printed stent-like structure comprising of 20 cylindrically wrapped 1mm diameter counter-rotating struts (Supplementary Fig. 13a). The target model - a cylindrical construct containing a trifurcated channel of 800µm diameter, was used to demonstrate printing of a convoluted negative feature within the presence of shadowing artifacts. Samples were prepared with 10% GelMA+0.1% LAP, with the addition of Cy5 to facilitate imaging for analysis. The occluding structure was placed within the vial and filled with the resin. The GelMa was thermally gelled in ice water, then the occluding mesh was scanned by using the light sheet as profilometer, as previously described. The resultant sparse surface reconstruction was then used to automatically align a reference 3D model of the occlusive surface. OSMO was used over 20 iterations to optimise the tomographic back projections and thus mitigate shadowing. After printing, samples were washed in warm PBS then imaged under light sheet for verification. Four samples - both uncorrected, and corrected, were analysed by segmenting out the interior channel using thresholding tools in ImageJ. The resultant models of the interior channels were analysed in CloudCompare (www.cloudcompare.org) with two representative samples shown in Supplementary Figure 12b. Additionally, the volumes of the segmented channels were also measured in Rhino3D (Supplementary Fig. 12c), as well as a metric for channel completeness, obtained by determining the height ratio of fully formed channels for each trifurcation, and the height of the entire construct (Supplementary Fig. 12d).

S14: Details for parametric models used for embedded extrusion printing

The parametric models, as depicted in Figure 4A, utilised raw positional data of the scanned toruses. These point clouds were processed in Grasshopper using the DENDRO plugin, where they were converted into a volumetric representation and subsequently used to generate a surface mesh of the extruded torus. The process of generating vascular-like channels towards to the torus was then performed as described in Supplementary Method **S7**, with the offset region (described by

step 4) instead being the surface mesh as described. A gaussian function was used to control the vessel radii along the length of the scaffold as a function of height.

S15: Cell isolation and expansion for femur and cartilage model

Equine tissues and cells were obtained from deceased donors, donated to science by their owner, according to the Institutional Animal Ethical Committee guidelines of Utrecht University. ACPCs and MSCs were isolated as previously described in literature[2]. Briefly, for ACPCs, macroscopically healthy cartilage from the metacarpophalangeal joint was harvested under sterile conditions, without damaging the tidemark. Cartilage was minced and consecutively digested in 0.2% pronase for 2 hours and 0.075% collagenase type II for 12 hours. The digested tissue was pelleted by centrifugation, suspended in serum-free DMEM, and plated in fibronectin-coated tissue culture plates, at a density of 500 cells/cm^2. Cells that did not adhere within 20 minutes were removed, whereas the attached cells were cultured in chondroprogenitor expansion medium (DMEM, supplemented with 10% v/v FBS, 0.2 mM ascorbic acid-2-phosphate, 100 U/mL penicillin, 100 µg/mL streptomycin, 1% v/v MEM non-essential amino acids solution and 5 ng/mL basic fibroblast growth factor (bFGF, Peprotech, UK)). After 6 days, colonies with more than 32 cells were collected, pooled and expanded until passage 4. Bone marrow aspirates were obtained from the sternum of equine donors. The mononuclear cell fraction was isolated using a Ficoll-paque density gradient (GE Healthcare, The Netherlands), after centrifugation for 30 minutes at 100g. After washing with PBS, the mononuclear cell fraction was centrifuged again at 300g for 10 minutes and plated on tissue culture plastic in MSC expansion medium (αMEM supplemented with 0.2 mM L-ascorbic acid 2-phosphate, 10% FBS, 100 U/mL penicillin with 100 µg/mL streptomycin and 1 ng/mL bFGF). Cells grown to passage 4 were used for this study.

S16: Femur-cartilage model microCT and staining

Constructs were imaged with a stereomicroscope after fixation in a 10% neutral buffered formalin solution. Samples were scanned with a microCT imaging system (Quantum FX; PerkinElmer, Waltham, MA, USA). Three minutes of scanning time was required per sample, at an isotropic voxel size of 42 µm resolution (voltage 90 kV, current 180 mA, field of view = 21 mm). Volumes of interest were segmented with a global threshold using image processing software 3D Slicer. 3D reconstructions were based on the micro-CT data and created using MeshLab. After scanning, samples were dehydrated in graded ethanol solutions (70–100%), cleared in xylene and

subsequently embedded in paraffin. 5 μm thick sections were cut using a microtome (Microm HM340E) and stained. Before staining, samples were deparaffinized with xylene and gradually rehydrated through decreasing ethanol solutions (100–70%). A triple staining of Weigert's haematoxylin (640490; Klinipath BV), fast green (FN1066522; Merck), and Safranin O (FN1164048213; Merck) was applied to identify cell nuclei, collagenous fibres and glycosaminoglycans, respectively. To detect mineralization, von Kossa staining was performed by incubating the sections with 1% silver nitrate (209 139, Sigma-Aldrich) directly under a light bulb for 2 hours. After washing the samples with 5% sodium thiosulfate (A17629, Alta Aesar, Haverhill, USA), they were counterstained with Mayer's haematoxylin. Images were taken with an Olympus BX51 microscope (Olympus DP73 camera, Olympus, Hamburg, Germany). Zonal distribution of the neo-synthesized extracellular matrix was correctly identified in the desired regions of the constructs, as reported in Figure S14A-E.

S17: Preparation of MSC spheroids

Human bone marrow-derived MSCs were isolated from bone marrow aspirates of consenting patients, as previously described[3]. Human bone marrow aspirates were obtained from the iliac crest of a patient (43 years old, female) that was receiving spondylodesis or hip replacement surgery. Isolation and distribution were performed in accordance with protocols approved by the Biobank Research Ethics Committee (isolation 08-001, distribution protocol 18-739, University Medical Centre Utrecht). Protocols used are in line with the principles embodied in the Declaration of Helsinki. MSCs were expanded in α-Modified Eagle Medium culture medium (α-MEM, Gibco, Life Technologies) supplemented with 10% FBS, 1% Penicillin/Streptomycin , 1% L-ascorbic acid-2-phosphate and 1 ng/ml basic fibroblast growth factor (bFGF; R&D Systems). Cells were cultured at 37°C, 5% CO2 and were used at passage 5. Medium was refreshed weekly. MSC spheroids were formed in a commercial microwell platform, AggreWell™400 (STEMCELL technologies, UK) and following manufacturer's instructions. MSCs were trypsinized with 0.25% Trypsin-EDTA (Gibco) and membrane-labelled for 30 min at 37°C with DiO or DiD (ThermoFischer Scientific). They were subsequently washed 1x with PBS and seeded at a concentration of 500 cells per μwell (6.0×10^5 cells/well) and spheroid formation occurred over 24h. MSC spheroids (diameter ≈ 150μm) were harvested according to the manufacturer's instructions. Aggregates were gently and thoroughly dislodged from the microwells and passed through a 40μm cell strainer placed on top a 50mL conical tube. Medium was dispensed once more

in the wells to wash them and collect leftover cells. The cell strainer was then inverted on top of a new 50mL conical tube and 3 mL of complete medium was passed through to collect the spheroids. After letting them settle for 3-5 min, the spheroids were resuspended in 10% w/v GelMA + 0.1% w/v LAP at a concentration of approximately 25 spheroids/mL. A first layer of GelMA containing DiD-labelled spheroids was put in the printing vial (8.8mm internal diameter) and left to thermally gelate with vigorous shaking (to ensure a homogeneous distribution of the spheroids in the resin), while immersed in cold water. After about 2 min, the second layer of GelMA containing DiO spheroids is placed on top and is similarly gelled while agitating.

S18: Idealised light sheet dose estimates over scanning period

A laser power meter (Coherent OP2-VIS, USA) was positioned at the focal point of the light sheet, and the resultant incident power was measured over a finite length L as bounded by the detector diameter (L=7.9mm). The measured powers were P_{450nm}=1.26mW, P_{532nm}=4.6mW, and P_{650nm}=3.73 mW. We modelled our light sheet intensity distribution $I(x,y)$ as gaussian (with beam quality factor $M^2 = 1.3$) along the *x-axis,* and as a top-hat profile of length L along the y-axis due to mean homogenisation by a Powell lens. This can be expressed as:

$$I(x,y) = \begin{cases} I_0 \, e^{-\frac{2x^2}{w_0^2}}, & |y| < \frac{L}{2} \\ 0, & otherwise \end{cases}$$

where ω_0 is the gaussian waist along x, L is the top-hat height, and I_0 corresponds to the on-axis peak intensity such that the total integrated power equals P. The power can be expressed as

$$P = \iint_{-\infty}^{\infty} I(x,y) dx \, dy$$

Which factorises into a gaussian integral along x and a top-hat along y

$$= I_o \int_{-\infty}^{\infty} e^{-\frac{2x^2}{\omega_0^2}} dx \int_{-\frac{L}{2}}^{\frac{L}{2}} dy$$

$$= I_0 \sqrt{\frac{\pi}{2}} L$$

Solving for I_0 yields:

$$I_0 = \frac{P\sqrt{2}}{\omega_0 L \sqrt{\pi}}$$

A script was then written in MATLAB to approximate the dosage over various scanning durations (discretised at 0.36° angular increments). The resultant radial dosage profile is shown in Supplementary Figure 15.

S19: Light dose distribution during light sheet imaging and cell compatibility

Our light sheet is equipped with 450nm, 532nm, and 650nn light sources (output: 40-50 mW), all in the visible range to minimize phototoxicity risks. Importantly, for the cell culture experiments involving the insulin release from pancreatic cells, and the osteal and chondral differentiation from stem cells, scanning was performed with the 650nm source, since the cells were stained with Vybrant DiD as indicated in the Methods section. Red light is being regarded generally as safer than shorter wavelengths, with previous reports of toxicity for doses >200 J/cm^2 [4], far beyond what delivered in our studies. In terms of light doses, we used scans lasting 75 or 150s for all our cell experiments. The calculated cumulative light dose distribution in every cross section parallel to the base of the vial for all the three lasers are reported in Supplementary Figure 15.

Due to our choice of using a radial light sheet setting, the delivered light dose peaks at the center of the printing vial, which is illuminated for the whole duration of the scan, and rapidly drops along the radius of the vial, where the voxels are illuminated only shortly during a scan as the light sheet sweeps the volume of the vat radially. Based on our model, outside of this 50µm-wide region, at 100µm away from the center of the vial the dose steeply drops to 14%, to further drop at 2.6% at 500 µm, to 1.4% at 1mm, to finally reach 0.09% at the inner border of the vial (7.5mm). Assuming that the lasers are operated at full power, and averaging the energy distribution in the beam profile cross-section, we obtain peak values of ~26, 90, and 66 J/cm^2 for the 450nm, 532nm, and 650nm lasers respectively, over a 75s scan. This value is confined at the center of the vial, and >99.7% (at 450nm), >96.8% (532nm), and >97.8% (650nm) of the circular cross-section of the vial receives a dose below 1 J/cm2, in the hundreds of mJ range, typical of conventional light sheet imaging[5]. To further modify the system and enable scanning while delivering lower light doses, it is important to note that the peripheral regions (>2mm away from the center of the vial) in our vat receive doses in the range 20-400mJ/cm^2, depending on the wavelength, and such doses were sufficient for feature detection, as shown by cellular structures resolved in these regions. In our design, we prioritized mechanical simplicity and ease of implementation, and therefore we opted for a radial light sheet, leveraging the rotating stage already available within the volumetric printer.

Changing the scanning procedure, however, GRACE could be performed using a more classical linear light sheet approach, in which every voxel of the samples is illuminated only once during a scan, therefore delivering a low, homogenous light dose across the sample, comparable to that found in the outer regions of the radial light sheet. Finally, the risk of phototoxicity can be further reduced by using pulsed illumination, or other light dose engineering strategies[6].

For our printing experiments involving insulin secretion and stem cell differentiation, in which the 650nm laser was used for scanning, we did not observe any abnormal behavior, and cells remained functional over 28 days of culture during the bone and cartilage differentiation assay. Nevertheless, to provide additional information, we assessed the effect of light exposure to the shorter wavelength laser (450nm) on cell viability, even though this specific laser line was not used in our functional cell printing experiments. Although its peak intensity is about 3-fold lower than the one from the 532nm laser, previous studies found that at comparable wavelengths, the maximum tolerable dose is about 5-fold lower in the blue range[4], and therefore we could test the cells in the worst-case scenario. MSC-laden GelMA hydrogels were subject to different light sheet scanning regimes, two "normal" illumination settings, used in this study (scanning time of 75s and 150s) and one condition with an extended scanning time (300s), more than what needed to perform GRACE, as control. Cell viability was assessed one day after exposure to detect photodamage, using a LIVE/DEAD viability kit (calcein AM/ethidium homodimer, Thermo Fisher Scientific). Cylindrical samples (6mm in diameter, 2mm height) were imaged with a fluorescence microscope, and the ratio of live and dead cells was counted from 3 randomly selected images per sample (number of samples per group, n=3). Non-imaged, non-printed samples, casted under a 405 nm light (1.4 mW/cm2, 3 minutes), as well as printed-only samples were used as controls. All samples displayed viability values >90% the day after exposure, with the few dead cells distributed seemingly randomly throughout the hydrogel samples indicating that our approach does not compromise cell viability (Supplementary Figure 16).

<u>S20: Dynamic Mechanical Analysis of GelMA hydrogels</u>

GelMA samples for mechanical testing were prepared at the concentrations used in this work: 10% w/v and 5%w/v GelMA supplemented with 0.1% (w/v) LAP. For each formulation, disk-shaped samples were prepared (diameter=6 mm, thickness=2 mm) either by volumetric printing, or via casting in a custom-made teflon mold and exposing the samples for 5 minutes under a 365 nm lamp delivering an intensity of 1 mW cm^{-2}). Both casted and printed GelMA disks were kept in

PBS in the incubator (37 °C; 5% CO2) overnight before testing to reach swelling equilibrium. The mechanical properties of the GelMA hydrogels were tested with the Dynamic Mechanical Analyzer (DMA Q800, TA Instruments) via a uniaxial, unconfined compression test. Samples (n = 4) were subjected to a load phase at 0.1 N min^{-1} up to 0.3 N. The compression modulus was calculated as the slope of the stress/strain curve in the 10–15% strain range. Results are reported in Supplementary Figure 17.

<u>S21: Bioprinting perfusable structures embedding high cell density features with GRACE</u>

MSC-laiden alginate particles were produced using the set-up previously described (Supplementary Methods S6). Briefly, MSCs (stained with the Vybrant DiD membrane dye to facilitate imaging) were suspended in a 2% w/v alginate solution in PBS, at a density of 200×10^6 cells/mL. The mix was extruded from the inner needle of the coaxial nozzle, under air flow running from the external nozzle, to produce droplets, which were collected in a bath filled with a100 mM solution of calcium chloride. The resulting cell-laden microgels were suspended and mixed into a gelatin methacryloyl resin (GelMA, 10% w/v), with 0.1% w/v LAP as a photoinitiator, and loaded into a vial of the tomographic printer. Upon light sheet scanning and using GRACE, channel networks were generated and printed, targeting individual spheres (Supplementary Fig. 18, Supplementary Video 4). Despite the potential of this approach, further improvements in the volumetric printing process would be highly beneficial. For example, in light-based bioprinting, achieving cell densities on the order of 10^8 cells/mL - typical of cell-dense tissues such as the heart or liver - remains a challenge when the suspension is homogeneously distributed within the resin vat, due to the strong light scattering of dense cell suspensions. Developing new strategies to overcome this limitation would significantly enhance the potential of volumetric printing, and by extension, of GRACE, for biomedical research.

<u>S22: Multiview reconstruction of complex surfaces using a digitally swept light sheet</u>

Reconstructing complex surfaces using light-sheet profilometry presents significant challenges when limited to a single polar sweep at a fixed central transverse position. This approach often fails to fully capture intricate volumes due to two primary factors: *i)* obstruction of the light sheet by the occluding object itself, and *ii)* inability to detect reflected or scattered light due to secondary obstructions within the scanned volume. To counter these limitations, we explored a multiview reconstruction strategy employing a digitally swept light sheet. This approach involves scanning

the object at multiple angles and transverse positions, significantly expanding the spatial sampling of the target volume. We implemented this into our setup by encoding different columns of 'on' pixels on the DMD to perform the transverse sweep, while maintaining the rotary stage's function for polar sweeps. This method generates 4D hyper-stacks $I(x, y, z, \theta)$ each containing fragments of surface profiles (Supplementary Fig. 20a).

We hypothesized that consolidating the contributions from each stack would yield a comprehensive occlusion map for shadow correction. To test this, we attempted to reconstruct the surface of a porous aluminium foam (Supplementary Fig. 20b). The aluminium foam was immersed in GelMA 10% with LAP 0.1% w/v. A scan was performed by digitally sweeping the light sheet column over each addressable horizontal position of the DMD (768 pixels in the horizontal direction), then performing an angular step of 2.88°, repeating this process over a full 360° to illuminate the volume at 125 angles. By employing a maximum intensity projection across all θ values for each spatial coordinate, a 3D image of the surface profile was partially reconstructed (Supplementary Fig. 20c), and a surface mesh generated (Supplementary Fig. 20d).

S23: Vial alignment and opportunities for further automation.

Reconstructing intricate geometries with volumetric printing requires precise alignment and careful positioning of the printing vial relative to both the axis of the rotating stage, and the optical projection axis7. This is also important for the GRACE workflow, as the success of the approach not only relies on the printing fidelity, but also the accuracy with which features can be mapped. Within our custom system, vial alignment and verticality are achieved by mounting the printing vial onto a 4-DoF positioner, comprising a tip/tilt kinematic V-clamp mount (Thorlabs KM100V/M) and an XY stage (Thorlabs DT12XY/M). Iterative alignment is achieved using a MATLAB routine that employs edge detection to provide an indication of the physical pitch/yaw and XY displacement of the vial, as well as the necessary corrections required. This is typically performed at the start of each print.

For future iterations of the GRACE workflow, recent advances in the state-of-the art, such as those outlined by Seymour et al.,[7] could be implemented within our workflow to perform comprehensive misalignment correction via the software. Specifically, by leveraging the volumetric imaging capabilities of GRACE, we envision inferring all degrees-of-freedom of misalignment within the vial by mapping and characterising features over a full 360° sweep. In practice, this would involve

acquiring polar light sheet scans many angles, detecting how fiducials or boundaries shift and rotate, and fitting those shifts to a geometric model that pinpoints net tilt, offset, or rotational errors. Once these misalignment parameters are known, new tomographic image sets could be generated. This holds true with the assumption of accurate alignment between the light sheet to the DMD projection axis - a condition which can be enforced by using a DMD-based light sheet, as described in Supplementary Methods S5. Through this approach, high-fidelity prints at improved reproducibility could be more easily achieved.

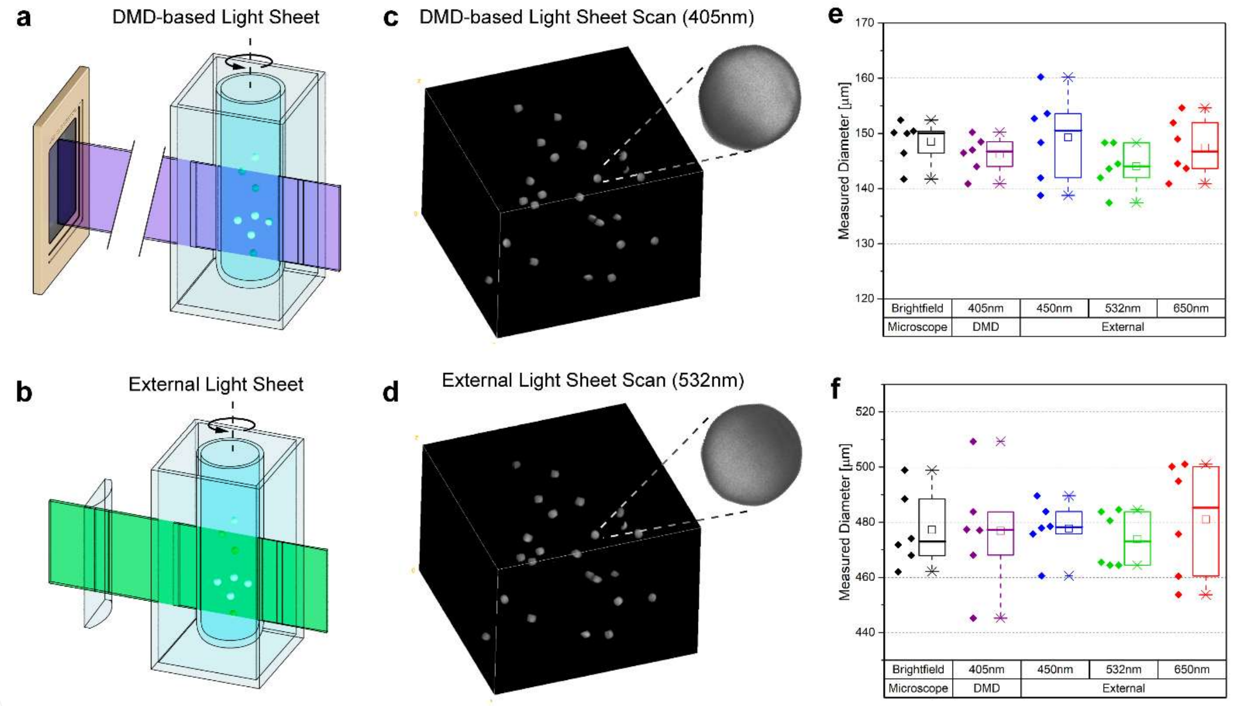

Supplementary Fig. 1.

Validation of using the DMD as a means of light sheet generation for volume scanning, by using polystyrene particles as reference features for benchmarking. **a,b)** A 3D representation of a DMD-generated light sheet formed by encoding a single pixel column onto the DMD, and illuminating with the volumetric printing source; and the externally generated light sheet used primarily throughout this work, respectively. **c,d)** An identical volume imaged then reconstructed from a scan using both the DMD-based, and external light sheets respectively. The volumes depicted contain the larger 425-500µm fluorescent beads. **e,f)** Graphs comparing the dimensions of fluorescent beads randomly distributed in gelatine, as measured by brightfield microscopy, 405nm

DMD generated light sheet, and the 450nm, 532nm, and 650nm external light sheet sources, for 425-500µm specified beads (boxes :25th and 75th percentile, whiskers 1.5 iqr, ANOVA, n=6, F=0.15 p=0.95, DF=29), and 125-150µm (boxes :25th and 75th percentile, whiskers 1.5 iqr, ANOVA, n=6, F=0.97, p=0.44, DF=34) beads, respectively. The graphs indicate no significant difference and comparable dimensional accuracy in all modalities. Moreover, particles smaller than the minimum detectable voxel size of our imaging system could not be detected, as tested with a batch of standard test particles with nominal average diameter=5µm.

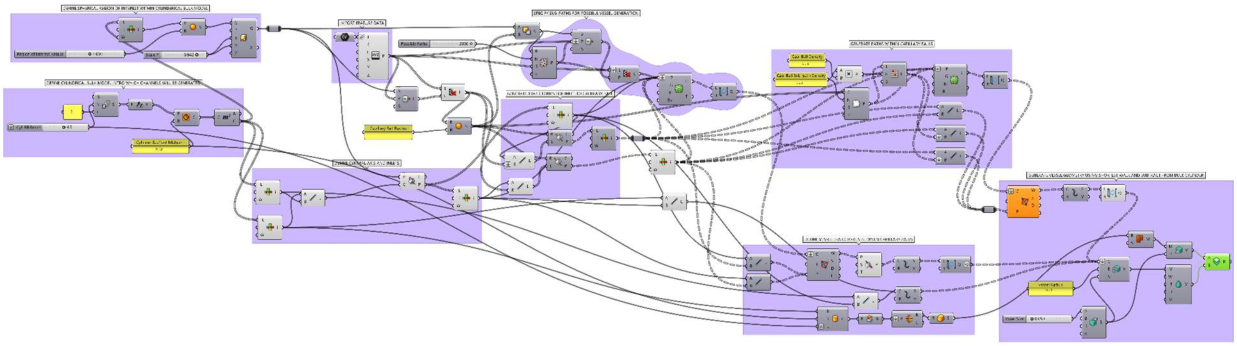

Supplementary Fig. 2.

A Grasshopper definition demonstrates the implementation of spherically wrapping channel network architectures within a bulk cylindrical scaffold, by which channels are generated towards- and around scanned features within a region of interest.

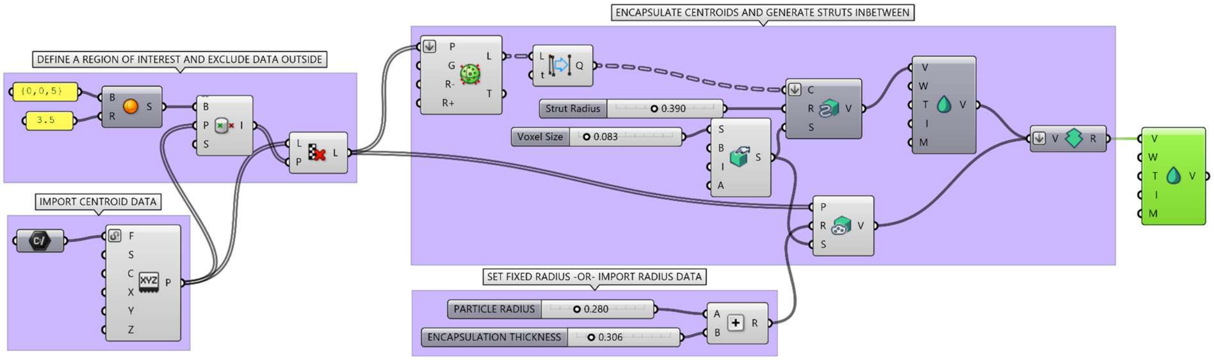

Supplementary Fig. 3.

Grasshopper definition flowchart demonstrates encapsulation with interconnection of scanned centroid data with struts-like geometries.

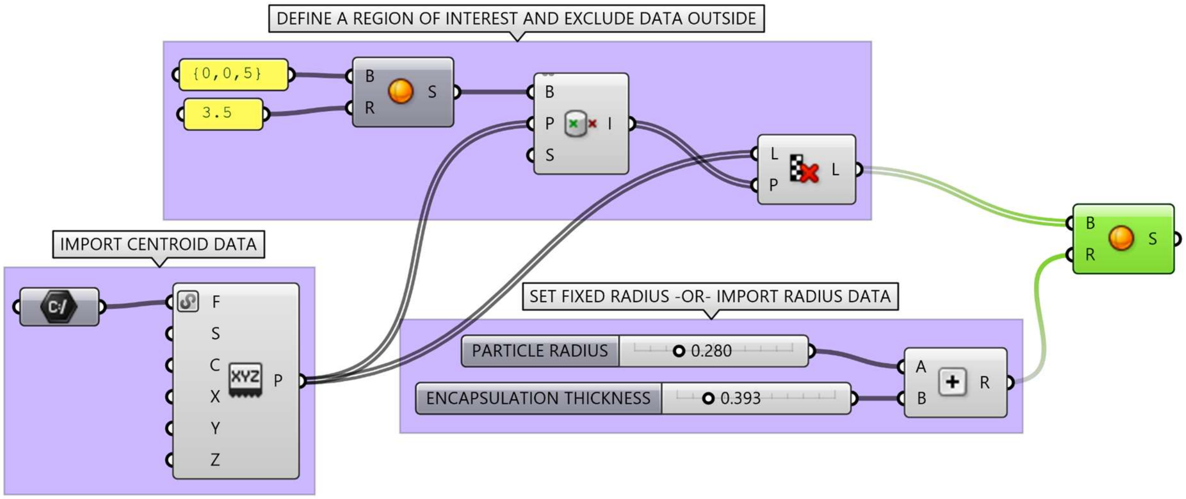

Supplementary Fig. 4.

Grasshopper definition flowchart demonstrating simple encapsulation of scanned centroid data with a spherical shell by means of placing spherical geometry in each coordinate.

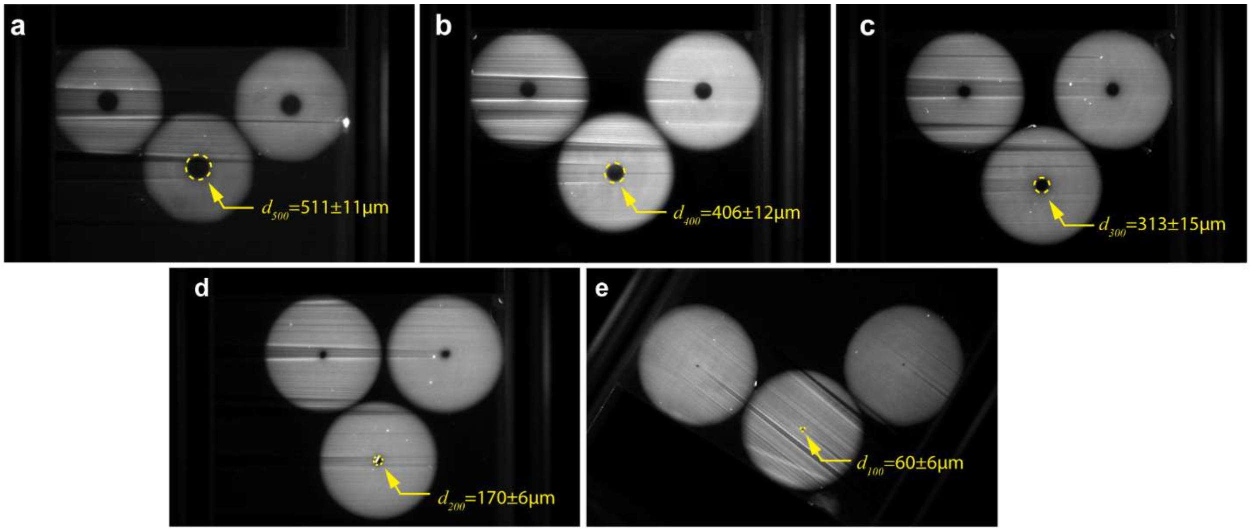

Supplementary Fig. 5.

Print resolution tests using vertically oriented tubular constructs with an external diameter of 4 mm and varying target internal diameters: **a)** d_{int} =0.5 mm, **b)** d_{int} =0.4 mm, **c)** d_{int} =0.3 mm, **d)** d_{int} =0.2 mm, and **e)** d_{int} =0.1 mm. Diametric accuracy begins to deviate below 0.2 mm, likely due to tighter tolerances on the optimal delivered dose.

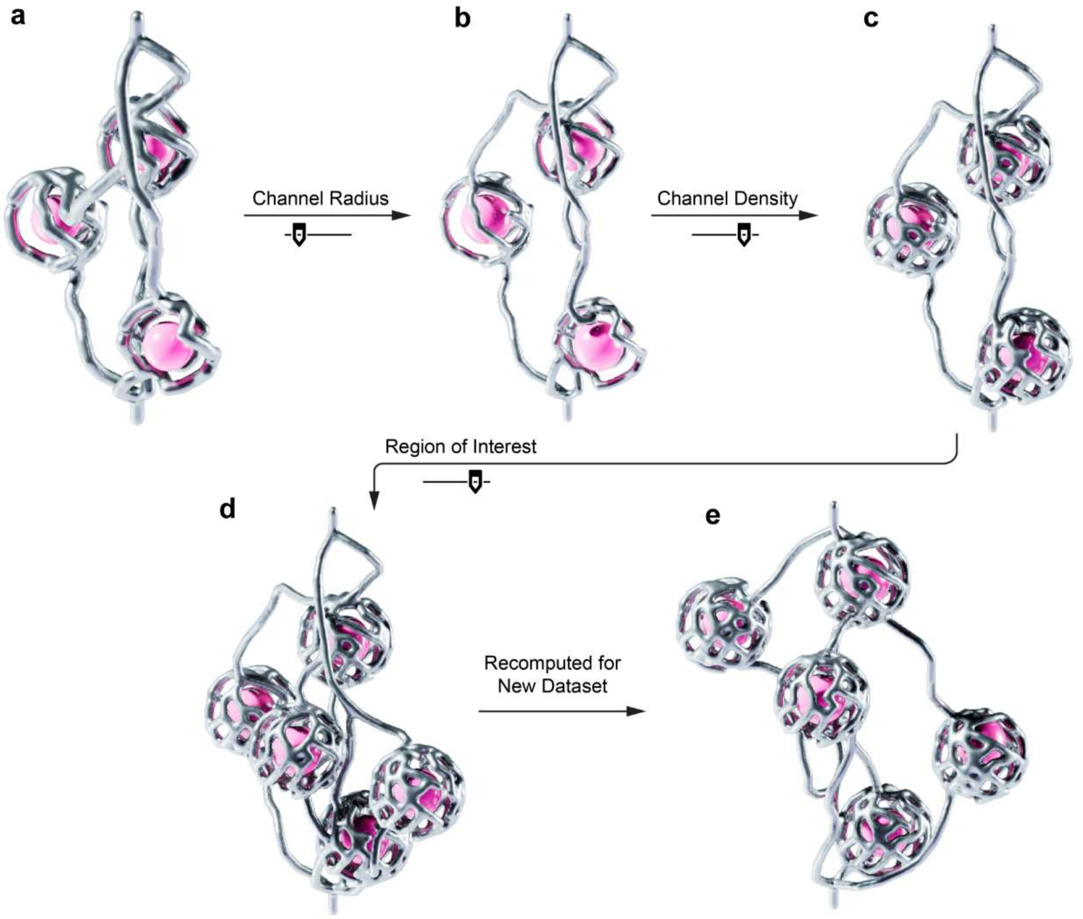

Supplementary Fig. 6.

Illustration highlighting the capability of parametric models to be effortlessly modified or adapted to different printing conditions or experimental requirements. **a)** Here, the parametric model generates spherically wrapping channel networks around detected features. On-the-fly changes to **b)** channel radius, **c)** channel density, and **d)** region of interest are accomplished through trivial modifications of parameters within the model. **e)** Also, upon repeating the GRACE workflow for a new sample, newly scanned features cause the model to be automatically recomputed, thus adapting to an entirely different dataset with no further intervention. By adjusting a small number of input parameters, structures can be rapidly refined, enabling efficient exploration of design space.

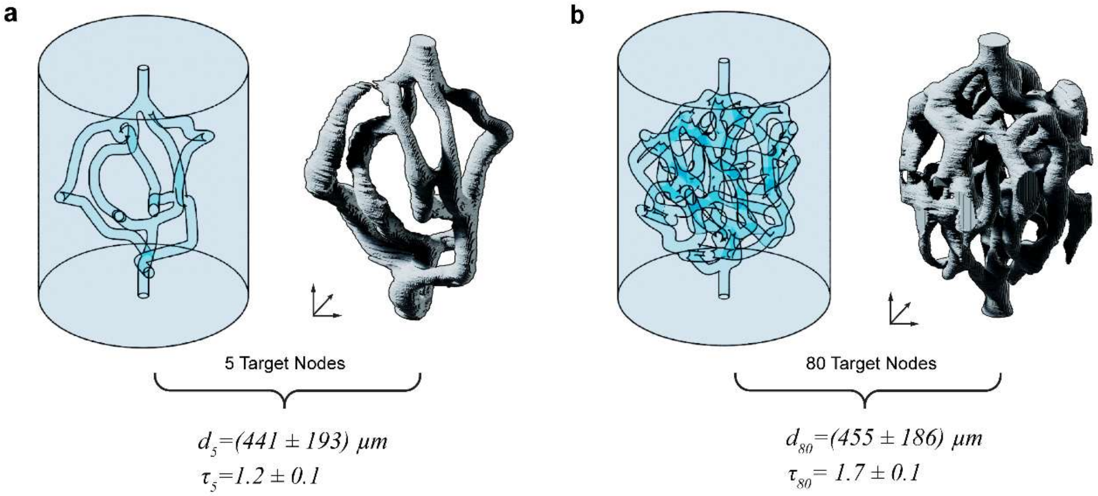

Supplementary Fig. 7.

Print tests of parametrically generated, convoluted channel networks with a 0.4 mm diameter, shown at two densities: **a)** low-density configuration with 5 target nodes; and **b)** high-density configuration with 80 target nodes. For both panels, the left image depicts a render of the target design to be printed, while the right image is a 3D light sheet reconstruction, segmenting out only the internal vessel network, coloured grey to facilitate visualization. Measured channel diameters *d* and tortuosity τ are shown for each print.

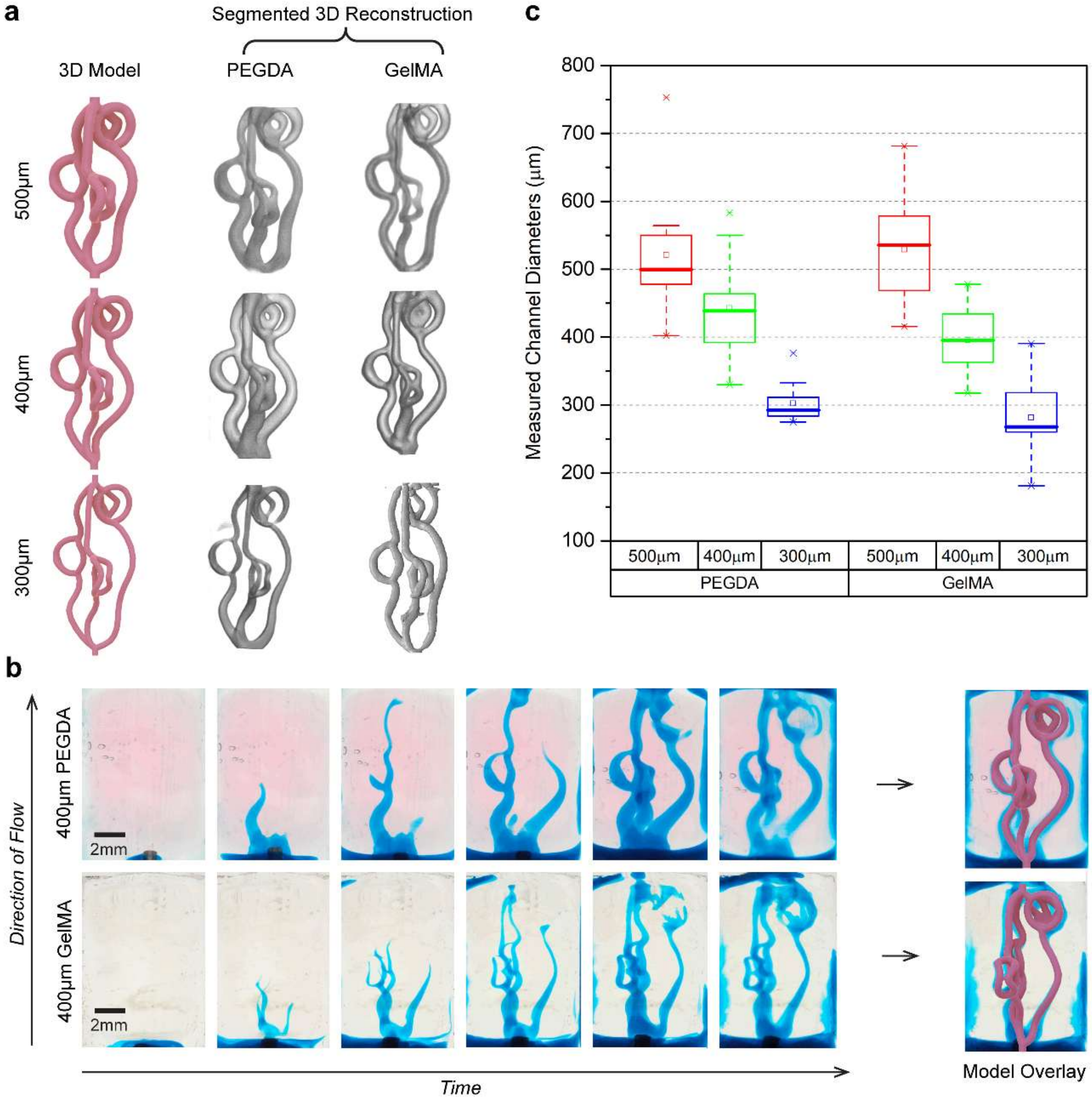

Supplementary Fig. 8.

A parametric design of a convoluted vessel network containing spherically wrapped channels was produced using GRACE from scanning a particle-laden sample. Thanks to the versatility of the parametric design, multiple STL files were produced adjusting the target vessel diameter (300, 400 or 500µm), without changing the overall design, to facilitate comparison across different prints. a) Light sheet 3D reconstruction of the printed channels (displayed in gray), produced via printing in a PEGDA or a GelMA resin. b) Time-lapse micrographs of the channel network being perfused with an alcian blue-stained solution, demonstrating full perfusability of the network. As the appearance of the channels in a 2D image is highly dependent on the angle of rotation of the sample, an overlay of the printed sample and the STL file at the corresponding orientation is also provided, to facilitate visualization. c) Quantification of the feature size (average diameter of the

printed vessels, boxes :25th and 75th percentile, whiskers 1.5 iqr). No significant differences were found between PEGDA and GelMA printed vessels at 500µm (t-test, number of measured vessels n=12, p=0.80), at 400µm (t-test, n=14, p=0.06), and at 300µm (t-test, n=12, p=0.27) .

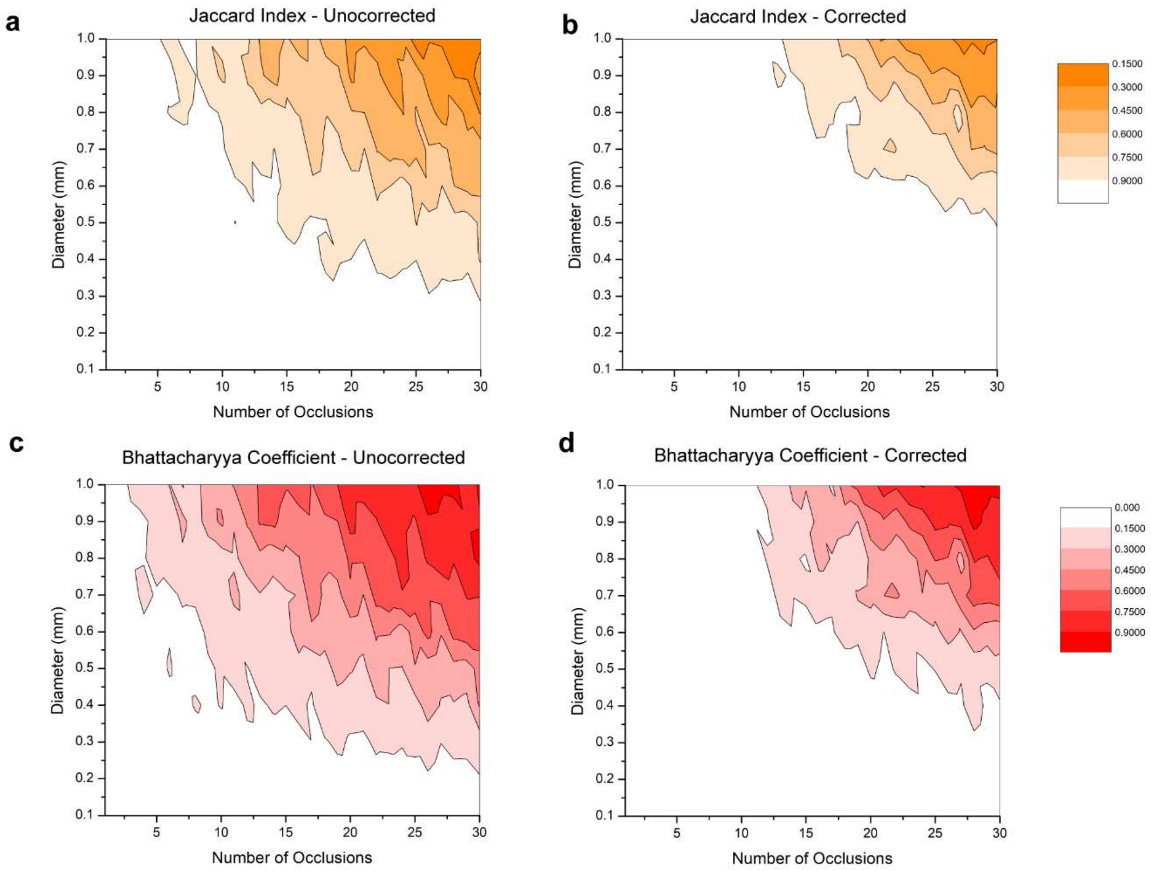

Supplementary Fig. 9.

Parametric sweep of randomly distributed circular occlusions within a 2D region spanning quantities between 1 to 30, and diameters between 0.1mm to 1mm. OSMO was used to optimize a cog-like 2D target geometry, with the resultant output quantified by **a,b)** the Jaccard index, and **c,d)** the Bhattacharyya coefficient.

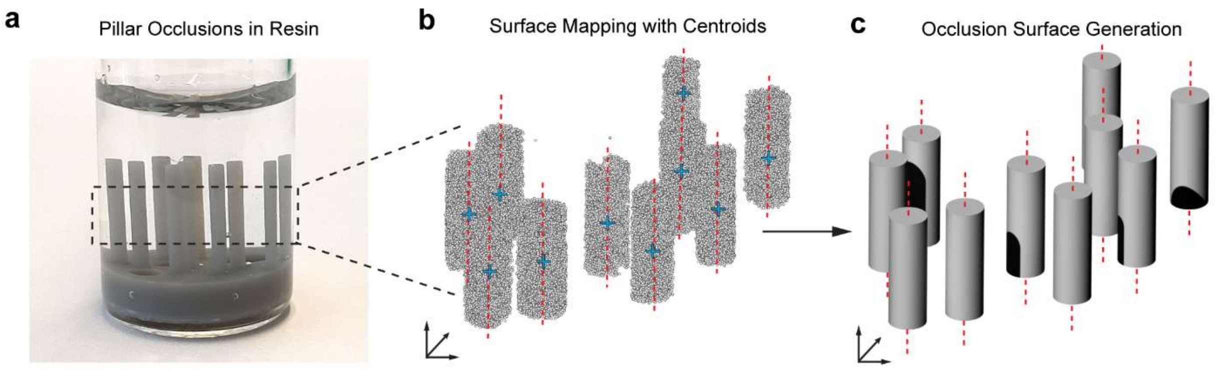

Supplementary Fig. 10.

a) Image of 3D printed pillars embedded in resin, used for demonstrating occlusion mapping and correction. **b)** Point cloud of the reconstructed mesh following polar light sheet profilometry scanning. **c)** Parametric generation of the geometry used to represent the occlusion following scanning and after applying a transformation.

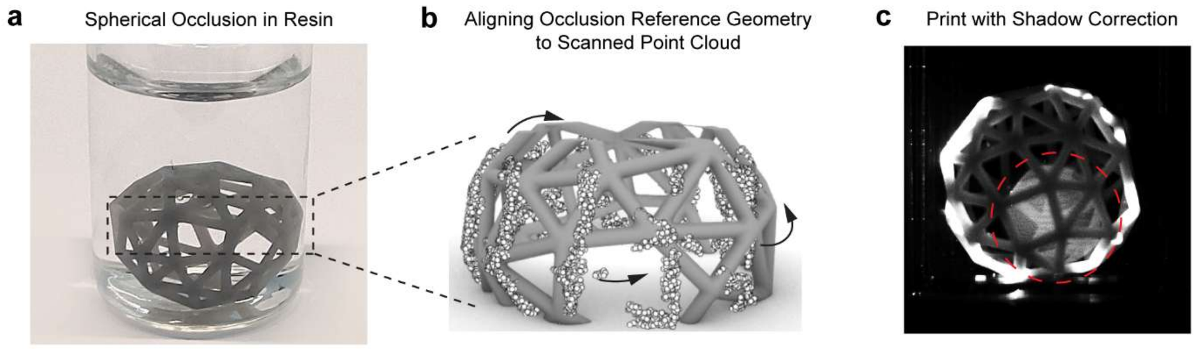

Supplementary Fig. 11.

a) Image of 3D printed spherical cage occluder embedded in resin. **b)** Point cloud of the reconstructed mesh following polar light sheet profilometry scanning, and the reference a priori reference mesh being aligned to it. **c)** Light sheet image of the shadow corrected target geometry (a sphere) printed inside a spherical cage after washing. The autofluorescence of the print is visible (denoted by a red circle) with the occlusive cage.

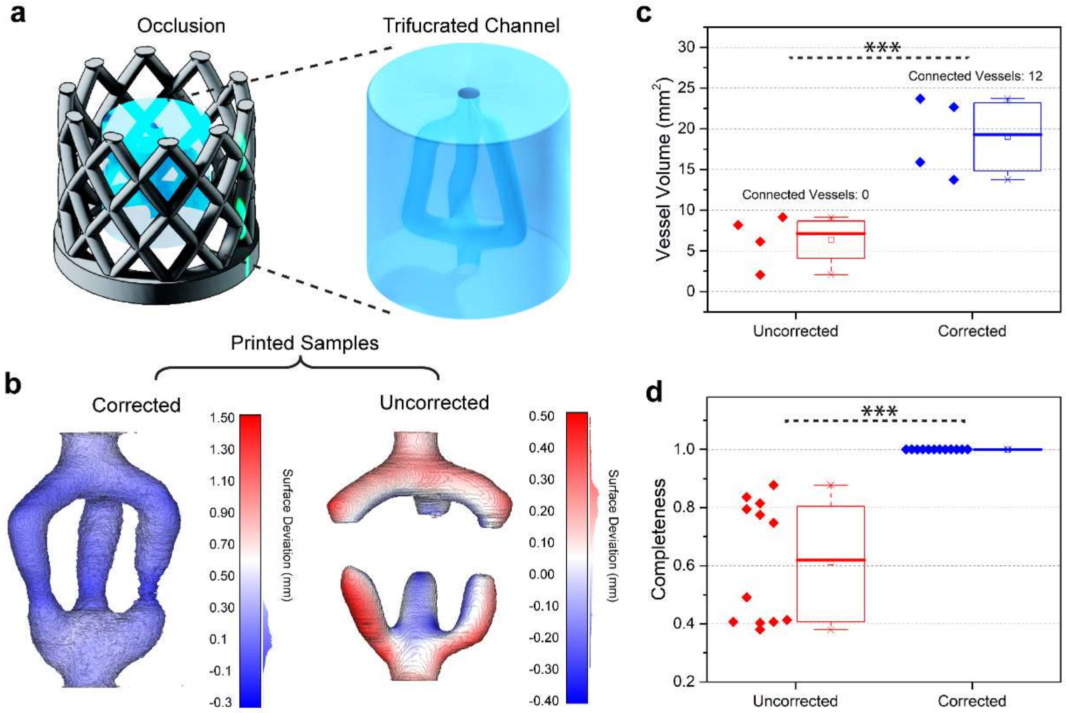

Supplementary Fig. 12.

a) GRACE printing of a three-branched vessel network in presence of a complex pattern of shadowing elements, representing a cylindrical cage with a stent-like structure. Although the mesh here has been used only as an occluding phantom, printing such vascular designs could be used to pattern perfusable structures within hydrogels, when these are coupled with mechanically strong, reinforcing scaffolds needed for load bearing applications. b) Representative 3D reconstructions of the vessel surface. The shadowing correction pipeline was able to reproduce the design in four out of four printing sessions, even in presence of the occluding mesh. Conversely, the uncorrected design always resulted in broken or incorrect reconstructions, in which the vessel lumen is clogged at different heights (hence, appearing empty in this 3D view), even if the printed parts had good fidelity compared to the reference model. Also for the corrected samples, the print fidelity of the channels was compared to the intended design, and the surface mismatch was colour-coded. The deviation maps and its associated histogram, show that the corrected design displays a peak of deviation of 0.1mm compared to the STL file, with the tail of the histogram reaching 0.3mm. This indicates the correction algorithm allows for reconstruction of objects within the light-occluding cage, a feature that would not be possible without GRACE. The challenge to deliver the correct light dose within the volume can affect print resolution, and this should be kept in mind when designing parts for specific applications. c) Quantification of the vessel volume for all prints (boxes

:25th and 75th percentile, whiskers 1.5 iqr, t-test, n=4, p=0.00497, DF=6). The lower vessel volume value for the uncorrected sample is a direct consequence of the fact that part of the object could not be resolved, and the vessels were clogged. d) completeness index calculated for each individual vessel branch (3 in each of the 4 samples), obtained by determining the height ratio of fully formed channels for each trifurcation, and the height of the entire construct. This index shows clearly that in no case it was possible to form completely a single vessel with the uncorrected projections, whereas 100% of the vessels were completed and connected when applying the GRACE-enabled shadow correction (boxes :25th and 75th percentile, whiskers 1.5 iqr, t-test, n=12, p<0.0001, DF=11).

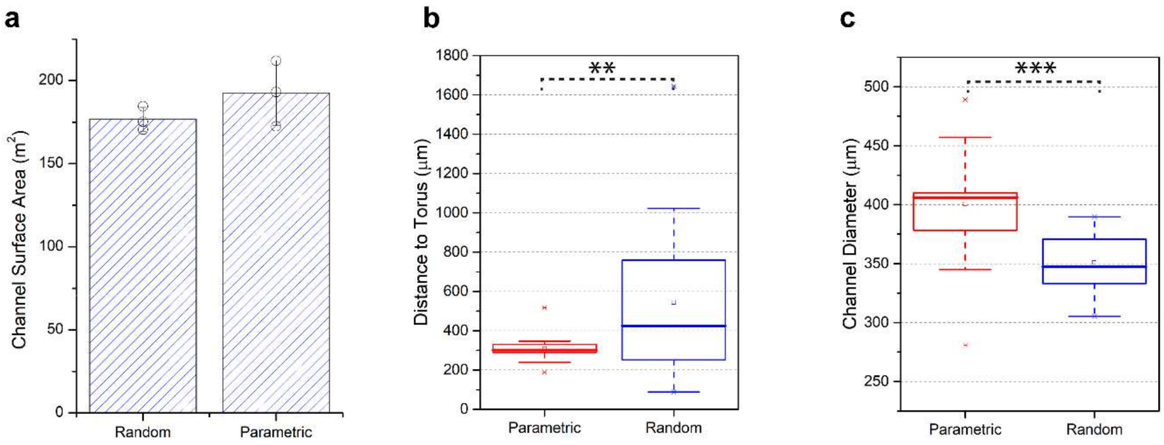

Supplementary Fig. 13.

a) Channel areas as determined via manual segmentation for scaffolds containing EmVP printed toruses. The graph indicates both random, and parametric GRACE-printed channels have equal surface areas as anticipated (mean±s.d., t-test, n=3, p=0.2622, DF=4). b) Measured distance of proximal channels from the torus at horizontal midplane for both the parametric and randomly designed channels (boxes :25th and 75th percentile, whiskers 1.5 iqr, t-test, n=18, p=0.016, DF=34). c) Measured diameters of proximal channels around the torus at the horizontal midplane for both the parametric and randomly designed channels (boxes :25th and 75th percentile, whiskers 1.5 iqr, t-test, n=21, p=7×10-5, DF=40).

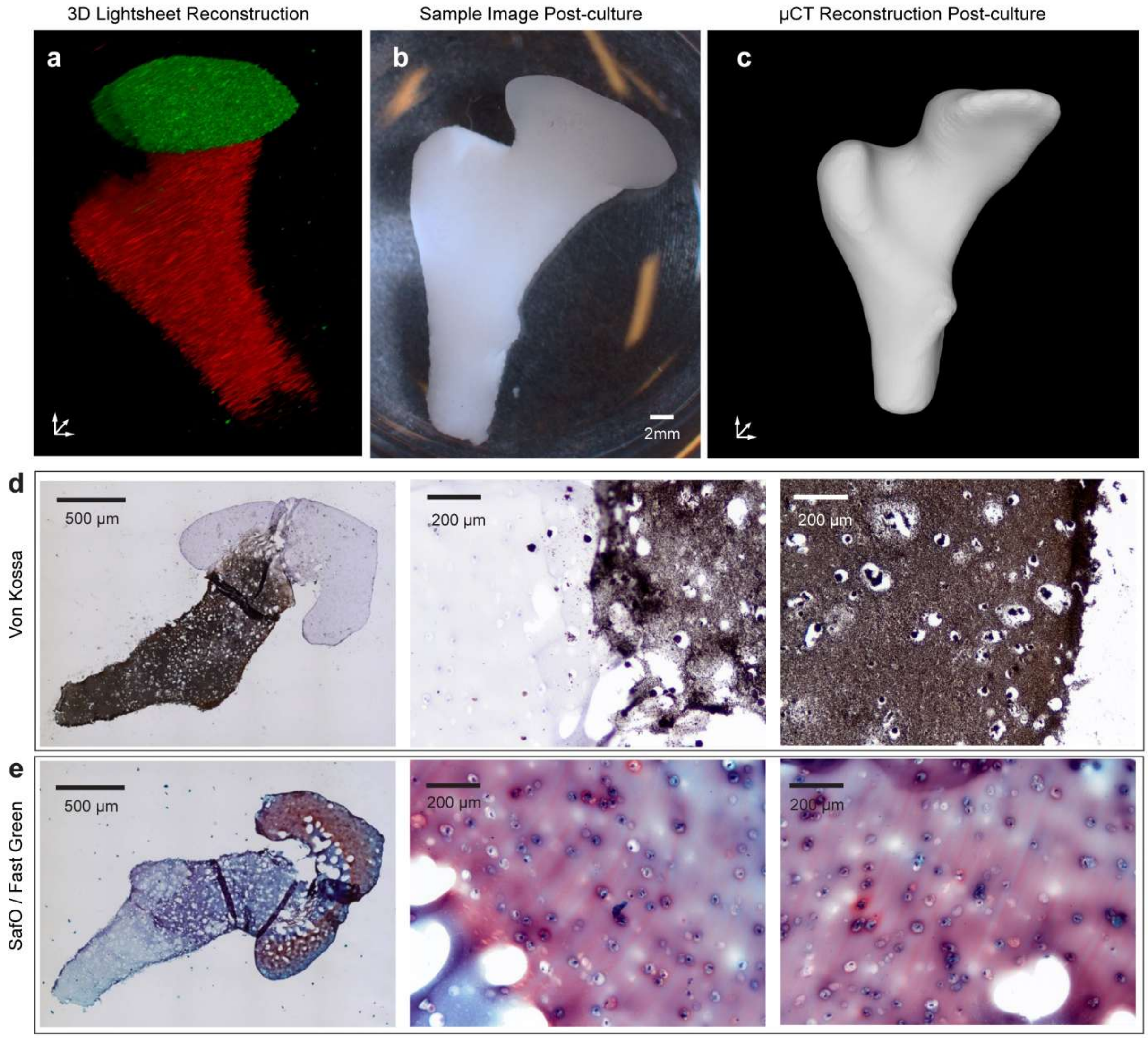

Supplementary Fig. 14.

a) Fluorescence 3D light sheet reconstruction showing locations of cells in their correct positions. **b)** Image of sample post-culture; **c)** micro-CT reconstruct of sample post-culture; and **d,e)** histological sections showing differentiated femoral and osteal components. The first two images of panels D and E are also reported in the main text, Fig.4.

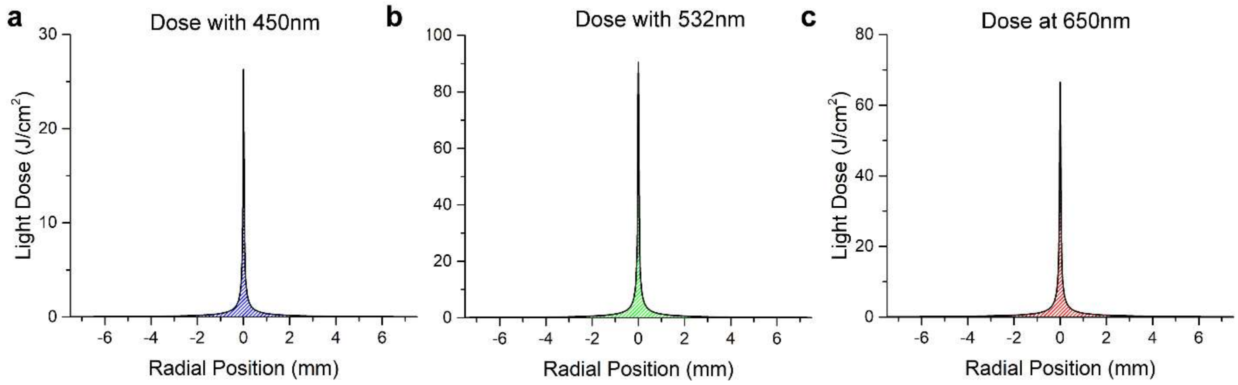

Supplementary Fig. 15.

Estimated light sheet dose within our system as a function of radial position within the printing volume. Dose estimates are performed for a upper-typical scanning duration of 150s, for **a)** the 450nm source, **b)** the 532nm source, and **c)** the 650nm source.

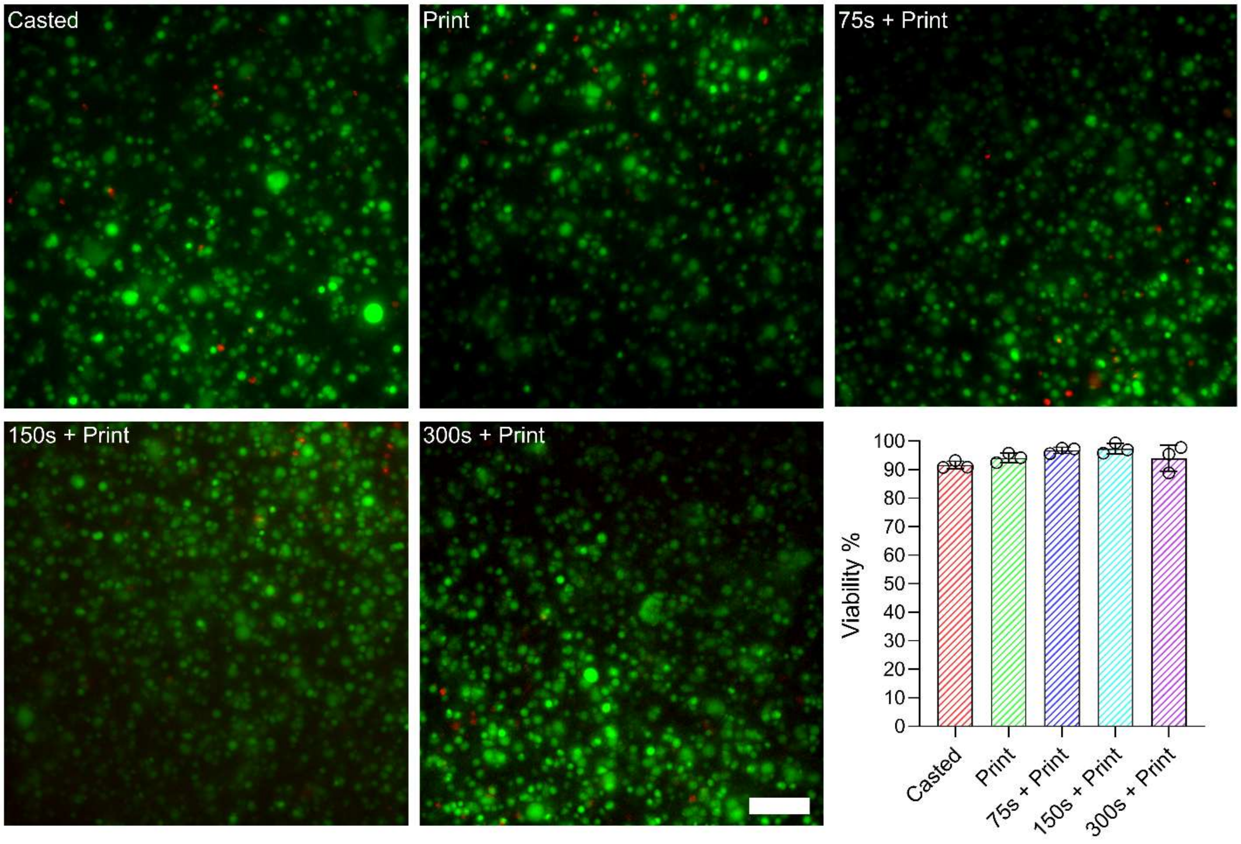

Supplementary Fig. 16.

Representative fluorescence microscopy images of the LIVE/DEAD staining for GelMA-encapsulated MSCs exposed to different light sheet scanning regimes. Samples produced via hydrogel casting or volumetric printing, but not imaged under the light sheet were used as control. For all samples, the ratio of living cells was above 90%, with no statistically significant differences detected (mean±s.d, ANOVA, n=3, p=0.0931, DF=14). Scalebar is 100µm.

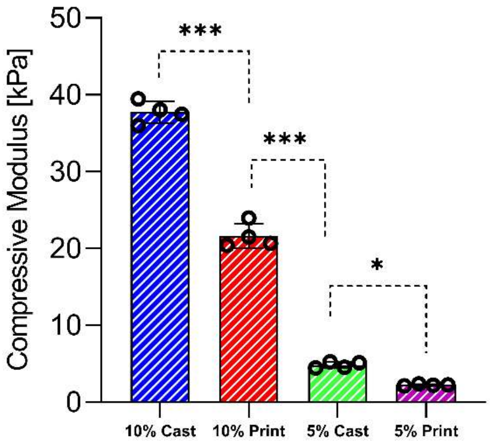

Supplementary Fig. 17.

Mechanical characterization under uniaxial, unconfined compression of GelMA hydrogels produced via volumetric printing and compared to casted controls. Hydrogels formed from precursor solutions at 5 and 10% w/v were produced and tested. These gels, used in this study, could be manipulated during culture, and they were mechanically stable under perfusion tests or cell culture. The compressive modulus of the hydrogels was found to be lower in volumetrically printed samples, compared to casted samples. (mean±s.d., ANOVA, n=4, ***p<0.0001, *p=0.0261, DF=12). This result is in line with what previously reported in the literature[8], as volumetric printing is performed stopping the photoexposure before the polymerization kinetics reaches its plateau, in order to avoid overcuring of small features, and therefore loss of shape fidelity. Higher crosslinking degree, and therefore mechanical properties, can only be reached via post-curing, when necessary. Partly cured GelMA resins post-volumetric printing have been previously demonstrated biocompatible and non-cytotoxic[9].

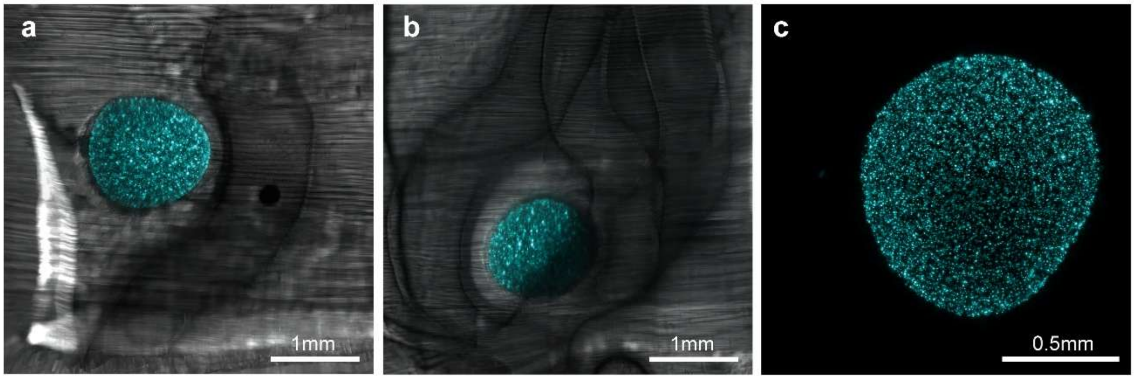

Supplementary Fig. 18.

Example of a GRACE printed structure by generating targeted features (channels) next to randomly distributed alginate spheres loaded with a concentration of 200 million MSCs/mL. **a,b)** Show two micrographs of two microspheres within different regions the same construct. The hydrogel sample was sliced with a razor blande, and imaged with a microscope. The pictures represent and overlay of the bright field, transmitted light image, showing the bulk of the gel and the printed channels running through it and of the fluorescence of the Vybrant DiD-stained cells (here digitally-colored in cyan). Scale bar = 1 mm. A full-thickness scan of the sample is provided in Supplementary Video 4. **c)** Close-up fluorescence microscopy image of a cell-laden alginate bead (scale bar = 500 μm).

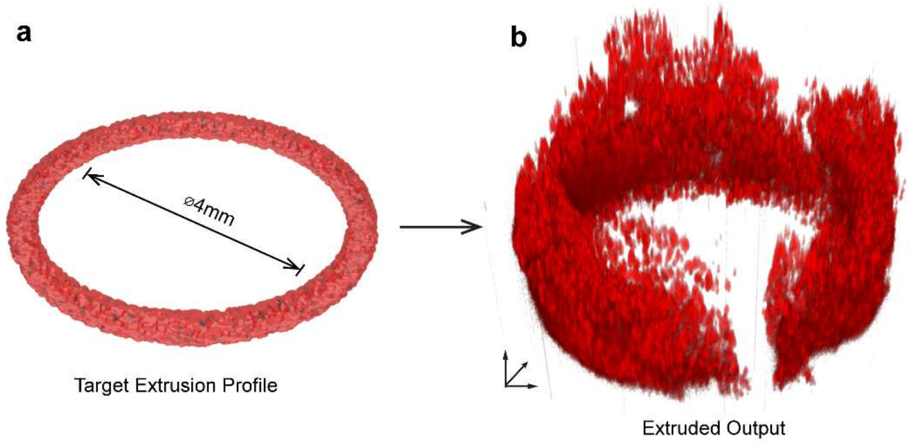

Supplementary Fig. 19.

a) Theoretical target extrusion profile when performing extrusion printing in a suspension bath (embedded printing) using a high cell concentration ink (iβ-cells, $5x10^7$ cells/mL in 2% w/v alginate), shown as a render of the CAD design, assuming the strut thickness is comparable to the extrusion nozzle diameter. b) Actual printing result typically obtained when extruding the same bioink in the GelMA suspension bath, clearly showing the deviation from the ideal shape, and the low shape fidelity common for such a high cell content ink.

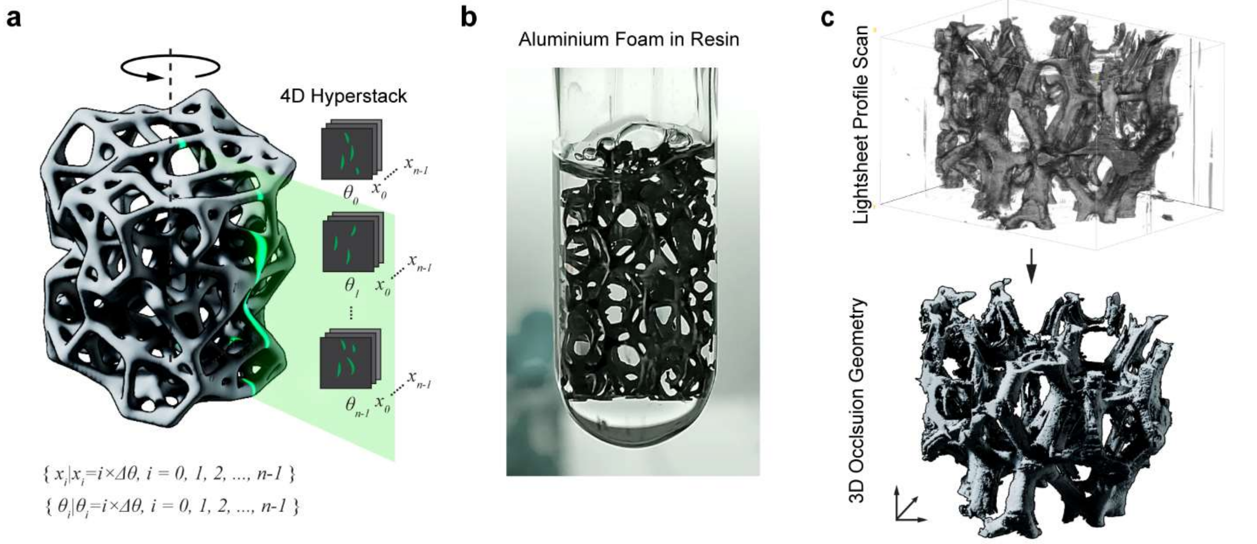

Supplementary Fig. 20.

Digitally swept lightsheet reconstruction resulting in 4D hyper-stack to reconstruct surfaces of porous aluminium foam. **a)** 3D representation of the rotational and translational profiling of the surface using a DMD-generated lightsheet; **b)** photograph of aluminium foam encapsulated in resin; **c)** processed 3D lightsheet reconstruction (top) and maximum projection vowelised surface (bottom).

Supplementary Video 1.

This video illustrates the process of tomographic volumetric 3D printing.

Supplementary Video 2.

Alginate particles were embedded within GelMA resin, then using GRACE, spherically wrapping channel networks were generated and printed. The movie shows the light sheet plane sweeping across the entirety of the sample, with highly concentric and convoluted channels clearly wrapping around the particles. The scan is performed with the bulk of the gel stained with Cy3.5, and therefore showing fluorescence in a green laser light sheet scan. The particles and the channels, non-fluorescent at that wavelength, appear in black.

Supplementary Video 3

A fluorescent particle is embedded in gelatin. GRACE is used to map its position within the vial, then selectively illuminates only this particle by projecting a thin beam of light. The tracking of the particle as it is rotated is visible within the video.

Supplementary Video 4

Series of light sheet cross-sections, showing GRACE-printed channels that were generated in presence of alginate microspheres encapsulating cells at a density of 200 million cells per mL (MSC, DiD stained). The video shows the channels (empty and open) in black, and the cell-laden hydrogel spheres lighting up when hit by the laser of the light sheet, due to the fluorescence emission of the DiD dye.

Supplementary Video 5

Series of light sheet cross-sections, showing GRACE-printed channels, formed in presence of a stent-like cage as occluding feature. The video shows the channels interconnectivity and their open lumen.

Supplementary References

1. Chen, Y. *et al.* Low-cost and scalable projected light-sheet microscopy for the high-resolution imaging of cleared tissue and living samples. *Nat. Biomed. Eng.* **8**, 1109 (2024).
2. Levato, R. *et al.* The bio in the ink: cartilage regeneration with bioprintable hydrogels and articular cartilage-derived progenitor cells. *Acta Biomaterialia* vol. 61 41–53 (2017).
3. Longoni, A. *et al.* Endochondral Bone Regeneration by Non-autologous Mesenchymal Stem Cells. *Front. Bioeng. Biotechnol.* **8**, 651 (2020).
4. Schneckenburger, H. *et al.* Light exposure and cell viability in fluorescence microscopy. *J. Microsc.* **245**, 311 (2012).
5. Power, R. M. & Huisken, J. A guide to light-sheet fluorescence microscopy for multiscale imaging. *Nat. Methods* **14**, 360 (2017).
6. Boudreau, C. *et al.* Excitation light dose engineering to reduce photo-bleaching and photo-toxicity. *Sci. Rep.* (2016) doi:10.1038/srep30892.
7. Seymour, G. & McLeod, R. R. Detection and correction of misalignment for high fidelity volumetric additive manufacturing. *Opt. Express* **33**, 6894–6908 (2025).
8. Cook, C. C. *et al.* Highly Tunable Thiol-Ene Photoresins for Volumetric Additive Manufacturing. *Adv. Mater.* **32**, (2020).
9. Bernal, P. N. *et al.* Volumetric Bioprinting of Organoids and Optically Tuned Hydrogels to Build Liver-Like Metabolic Biofactories. *Adv. Mater.* **34**, 2110054 (2022).
