# Supplementary material for: Adaptive and context-aware volumetric printing
Source: Nature. 2025 Sep 3;645(8079):108–14. doi: 10.1038/s41586-025-09436-7 (PMC12408377; doi:10.1038/s41586-025-09436-7)
Supplement: Supplementary file 2 — Reporting Summary [file 41586_2025_9436_MOESM2_ESM.pdf]

nature portfolio

Corresponding author(s): Riccardo Levato
Last updated by author(s): Jun 29, 2025

Reporting Summary

Nature Portfolio wishes to improve the reproducibility of the work that we publish. This form provides structure for consistency and transparency in reporting. For further information on Nature Portfolio policies, see our Editorial Policies and the Editorial Policy Checklist.

Statistics

For all statistical analyses, confirm that the following items are present in the figure legend, table legend, main text, or Methods section.

n/a	Confirmed	
☐	☒	The exact sample size (*n*) for each experimental group/condition, given as a discrete number and unit of measurement
☐	☒	A statement on whether measurements were taken from distinct samples or whether the same sample was measured repeatedly
☐	☒	The statistical test(s) used AND whether they are one- or two-sided<br>*Only common tests should be described solely by name; describe more complex techniques in the Methods section.*
☒	☐	A description of all covariates tested
☐	☒	A description of any assumptions or corrections, such as tests of normality and adjustment for multiple comparisons
☐	☒	A full description of the statistical parameters including central tendency (e.g. means) or other basic estimates (e.g. regression coefficient) AND variation (e.g. standard deviation) or associated estimates of uncertainty (e.g. confidence intervals)
☐	☒	For null hypothesis testing, the test statistic (e.g. *F*, *t*, *r*) with confidence intervals, effect sizes, degrees of freedom and *P* value noted<br>*Give P values as exact values whenever suitable.*
☒	☐	For Bayesian analysis, information on the choice of priors and Markov chain Monte Carlo settings
☒	☐	For hierarchical and complex designs, identification of the appropriate level for tests and full reporting of outcomes
☒	☐	Estimates of effect sizes (e.g. Cohen's *d*, Pearson's *r*), indicating how they were calculated

Our web collection on statistics for biologists contains articles on many of the points above.

Software and code

Policy information about availability of computer code

Data collection	The following softwares were used for data collection:<br>- Matlab 2023a (MathWorks, USA)<br>- Rhino 7 (Robert McNeel & Associates, USA)
Data analysis	Statistical analyses for experiments involving shadow correction were conducted using OriginPro 8.5 (OriginLab, USA), while Prism 9 software (GraphPad Inc., USA) was used for the EmVP experiments.

For manuscripts utilizing custom algorithms or software that are central to the research but not yet described in published literature, software must be made available to editors and reviewers. We strongly encourage code deposition in a community repository (e.g. GitHub). See the Nature Portfolio guidelines for submitting code & software for further information.

Data

Policy information about availability of data

All manuscripts must include a data availability statement. This statement should provide the following information, where applicable:

- Accession codes, unique identifiers, or web links for publicly available datasets
- A description of any restrictions on data availability
- For clinical datasets or third party data, please ensure that the statement adheres to our policy

Supplementary Information is available for this paper. Correspondence and requests for materials should be addressed to r.levato@uu.nl. Animal cells used for this study and codes for the parametric printing process are available under a research agreement with Utrecht University.

Research involving human participants, their data, or biological material

Policy information about studies with human participants or human data. See also policy information about sex, gender (identity/presentation), and sexual orientation and race, ethnicity and racism.

Reporting on sex and gender	not applicable
Reporting on race, ethnicity, or other socially relevant groupings	not applicable
Population characteristics	not applicable
Recruitment	not applicable
Ethics oversight	not applicable

Note that full information on the approval of the study protocol must also be provided in the manuscript.

Field-specific reporting

Please select the one below that is the best fit for your research. If you are not sure, read the appropriate sections before making your selection.

☒ Life sciences ☐ Behavioural & social sciences ☐ Ecological, evolutionary & environmental sciences

For a reference copy of the document with all sections, see nature.com/documents/nr-reporting-summary-flat.pdf

Life sciences study design

All studies must disclose on these points even when the disclosure is negative.

Sample size	Sample size was at least n>=3 for all experiments. No power analysis was performed ahead of the study. The choice is based on the previous experiment in which the selected size were shown to be sufficient to observe statistically significant differences, for the magnitude of effect we typically observe. For all cell culture tests, sample size was either 3 or 4. For printing tests (cell free), samples were printed in 3 replicates. Monte Carlo simulations (data analyzed and reported in figure 2b) were repeated 12 times.
Data exclusions	no exclusion
Replication	Attempts at replications in our lab were successful
Randomization	not applicable
Blinding	not applicable. Investigators could not be blind when extracting data from imaging information (the images clearly reveal which experimental group is being analyzed).

Reporting for specific materials, systems and methods

We require information from authors about some types of materials, experimental systems and methods used in many studies. Here, indicate whether each material, system or method listed is relevant to your study. If you are not sure if a list item applies to your research, read the appropriate section before selecting a response.

Materials & experimental systems

n/a	Involved in the study
☒	☐ Antibodies
☐	☒ Eukaryotic cell lines
☒	☐ Palaeontology and archaeology
☒	☐ Animals and other organisms
☒	☐ Clinical data
☒	☐ Dual use research of concern
☒	☐ Plants

Methods

n/a	Involved in the study
☒	☐ ChIP-seq
☒	☐ Flow cytometry
☒	☐ MRI-based neuroimaging

Eukaryotic cell lines

Policy information about cell lines and Sex and Gender in Research

Cell line source(s)

We used iB cells, originally developed by the Fussenegger group at ETH Zurich, who also provided these cells to us. The papers describing how the line is produced are listed in the Methods section (specificlaly, reference 27: https://onlinelibrary.wiley.com/doi/10.1002/smll.202101939).

Authentication

cells are capable to release insulin (measured by ELISA) and nanoLuc (measured via luminescent assays, NanoLuc Luciferase Kit (Promega Corporation, USA)) as reporter.

Mycoplasma contamination

not tested

Commonly misidentified lines
(See ICLAC register)

iB cells is a line derived from engineering 1.1E7 with synthetic gene circuits to release insulin and nanoLuc as reporter. While 1.1E7 a cell line listed in the ICLAC register, iB is a new line (see above), described by the Fussenegger lab at ETH Zurich. In this work, iB has been used to assess its capacity to release insulin and nanoLuc as a function of the bioprinted vascular geometries. The cells were able to perform this function, and therefore to be safely used for our experiment.

Plants

Seed stocks

not applicable

Novel plant genotypes

not applicable

Authentication

not applicable
